# Utilisation et efficacité des moustiquaires imprégnées d'insecticide à longue durée d'action (MILDA) dans le sud du Cameroun

**DOI:** 10.48327/mtsi.v6i2.2026.838

**Published:** 2026-04-08

**Authors:** Roméo Serge MBONGUE, Jean Arthur MBIDA MBIDA, Michel Léger OFFONO ENAMA, Pasma MÂCHE NKOUANDOU, Mathias NWAHA, Odette Étoile NGO HONDT, Francis Noël NOPOWO TAKAP, Williefred DONGMO TEKAPI, Rachel NGAHA, Wolfgang EKOKO EYISAP, Parfait AWONO AMBEME, Patrick AKONO-NTONGA

**Affiliations:** 1 Département des sciences biomédicales, Faculté des sciences, Université de Bertoua, B.P.416, Bertoua, Cameroun; 2 Département de biologie et physiologies des organismes animaux, Faculté des sciences, Université de Douala, B.P. 24157, Douala, Cameroun; 3 Département de sciences biologiques des organismes vivants, Faculté des sciences, Université de Garoua. B.P. 346, Garoua, Cameroun; 4 Département des sciences biologiques, Faculté des sciences, Université de Yaoundé. B.P. 812 Yaoundé, Cameroun; 5 Institut de recherche de Yaoundé (IRY), Organisation de coordination pour la lutte contre les endémies en Afrique Centrale (OCEAC), P.O. Box 288, Yaoundé, Cameroun; 6 Laboratoire de chimie, Université de Douala, P.O. Box 2701, Douala, Cameroun

**Keywords:** MILDA, Utilisation, Bio-efficacité, Intégrité physique, Kribi, Région du Sud, Cameroun, Afrique subsaharienne, LLIN, Use, Bioefficacy, Physical integrity, Kribi, South Region, Cameroon, Sub-Saharan Africa

## Abstract

**Contexte:**

Les moustiquaires imprégnées d'insecticide à longue durée d'action (MILDA) sont largement utilisées en Afrique subsaharienne pour réduire la transmission du paludisme. Leur durée de vie opérationnelle dans les conditions de terrain dépend de nombreux facteurs. Cet article présente certains de ces facteurs associés à la dégradation de l'efficacité des MILDA en milieu urbain et rural à Kribi au sud du Cameroun.

**Méthodes:**

L’étude, réalisée en 2019, visait à évaluer l’utilisation et l'efficacité résiduelle des moustiquaires contre deux souches d'*Anopheles gambiae* s.l., une souche locale avec un statut de sensibilité aux insecticides inconnu et une souche de laboratoire de référence sensible.

**Résultats:**

Au total, 540 ménages ont été enquêtés. La couverture en MILDA était similaire entre les zones rurale (69,0 %) et urbaine (68,6 %). Le nombre d'enfants de moins de 5 ans utilisant des MILDA (88,6 % : 651/735) était comparable dans les deux contextes. Les moustiquaires PermaNet 2.0 étaient plus dégradées que les autres marques dans la zone rurale, tandis que les moustiquaires Yorkool l’étaient davantage dans la zone urbaine. Les MILDA ayant subi plus de 20 lavages ont induit une mortalité de 21,6 % à 99,6 % pour la souche Kisumu et de 0,8 % à 76,5 % pour la souche sauvage. Celles lavées avec du savon en morceaux ont mieux conservé leurs propriétés létales par rapport à celles lavées avec des détergents corrosifs. De même, celles qui avaient séché à l'ombre avaient davantage conservé leurs propriétés létales que celles étendues au soleil.

**Conclusion:**

La population étudiée utilisait les MILDA particulièrement pour les enfants. La nature de fibre de la moustiquaire, les détergents ainsi que la lumière du soleil sont des facteurs qui influent sur l'effet insecticide des moustiquaires.

## Abstract (en)

**Background:**

Long-lasting insecticidal nets (LLINs) are widely used in sub-Saharan Africa to reduce the transmission of malaria. Their operational lifespan under field conditions depends on many factors. This article presents some of the factors associated with the decline in LLIN efficacy in urban and rural settings in Kribi, southern Cameroon.

**Methods:**

This study, conducted in 2019, aimed to evaluate the usage and residual efficacy of the nets against two strains of *Anopheles gambiae* s.l.: a local strain with an unknown insecticide susceptibility status and a sensitive reference laboratory strain.

**Results:**

A total of 540 households were surveyed. LLIN coverage was similar in rural (69.0%) and urban (68.6%) areas. The proportion of children under five years of age using LLINs was comparable in both settings (88.6%: 651/735). PermaNet 2.0 nets were more degraded than other brands in rural areas, while Yorkool nets were more degraded in urban areas. Mortality rates for the Kisumu strain ranged from 21.6% to 99.6% for LLINs that had been washed more than 20 times, and from 0.8% to 76.5% for the wild-type strain. Those washed with bar soap retained their lethal properties better than those washed with harsh detergents. Similarly, nets dried in the shade retained their lethal properties better than nets hung out in the sun.

**Conclusion:**

The study population used MILDAs, especially for children. The fiber composition of the nets, the type of detergent used, and exposure to sunlight are factors that influence the insecticidal efficacy of the nets.

**Figure:**
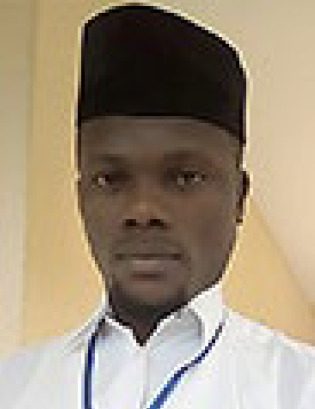

## Introduction

Le paludisme est l’une des maladies parasitaires les plus meurtrières dans le monde. En 2018, près de 228 millions de personnes ont contracté le paludisme parmi lesquelles environ 409 000 décès ont été notés [28]. L’Afrique subsaharienne est la zone la plus vulnérable. Au Cameroun, le paludisme, principale cause de morbidité et de mortalité, constitue un problème majeur de santé publique. En 2019, cette maladie a entraîné environ 11 000 décès notifiés et impacte négativement l’économie de la cellule familiale et de la nation toute entière [21]. Les cibles les plus vulnérables sont les femmes enceintes et les enfants de moins de cinq ans [22]. Le gouvernement camerounais a défini avec le Programme national de lutte contre le paludisme (PNLP) un certain nombre d’orientations stratégiques, dont la prévention par l’utilisation des moustiquaires imprégnées d’insecticide est la principale [21].

L’utilisation des moustiquaires, d’abord contre les nuisances culicidiennes, ensuite pour réduire la transmission des agents infectieux est une pratique très ancienne [17]. Dans le cadre de la lutte antipaludique, les premiers essais réalisés dans différents pays ont permis de démontrer l’impact des moustiquaires imprégnées d’insecticide sur l’incidence de la maladie [17]. Cependant, ces moustiquaires présentaient l’inconvénient de devoir être ré-imprégnées régulièrement, au moins une fois par an [18]. Des moustiquaires imprégnées d’insecticide à longue durée d’action (MILDA) ont été produites comme alternative à la ré-imprégnation. Ce sont des moustiquaires prétraitées industriellement par des procédés spécifiques qui leur permettent d’être efficaces après au moins 20 lavages et de conserver leurs propriétés insecticides pendant 3 à 5 ans en usage normal [26]. Un insecticide de la famille des pyréthrinoïdes, incorporé ou enrobé dans les fibres en polyester ou en polyéthylène pendant la fabrication, se libère lentement et migre vers la surface de la moustiquaire, provoquant un effet dissuasif, répulsif ou excito-répulsif. À des niveaux élevés de couverture, les avantages de l’utilisation des MILDA à l’échelle communautaire ont été démontrés. Au-delà de la protection personnelle, un effet de masse sur la population des vecteurs est observé.

Les MILDA constituent l’un des fers de lance de la lutte antivectorielle (LAV). En Afrique subsa-harienne, la proportion des populations ayant accès à une MILDA est passée de moins de 2 % en 2000 à 67 % en 2015. Une couverture universelle est atteinte dans certains pays [26]. La question essentielle de leur durée de vie réelle (intégrité physique et bio-efficacité) reste posée. Il existe un déficit d’investigation sur la durée de vie fonctionnelle et la variation des performances entre les différentes MILDA dans diverses conditions d’utilisation. Des études menées en Ouganda ont montré que 45 à 78 % des moustiquaires étaient endommagées après une année d’utilisation [13]. Au Kenya et au Bénin, une détérioration plus rapide que prévue de leur bio-efficacité a été notée, soulevant des questions sur leur durée de vie effective [2-6,11]. Dans une étude menée au Laos, environ 40 % des moustiquaires étaient endommagées après 2 à 3 ans d’utilisation [30]. La localité de Kribi dans le Sud-Cameroun compte parmi celles ayant bénéficié d’une distribution de masse de MILDA en 2019. La présente étude fait le point sur le degré d’intégrité physique et la bio-efficacité des MILDA au bout de trois ans de leur utilisation par les ménages de la localité de Kribi.

## Matériel et méthodes

**Figure 1:**
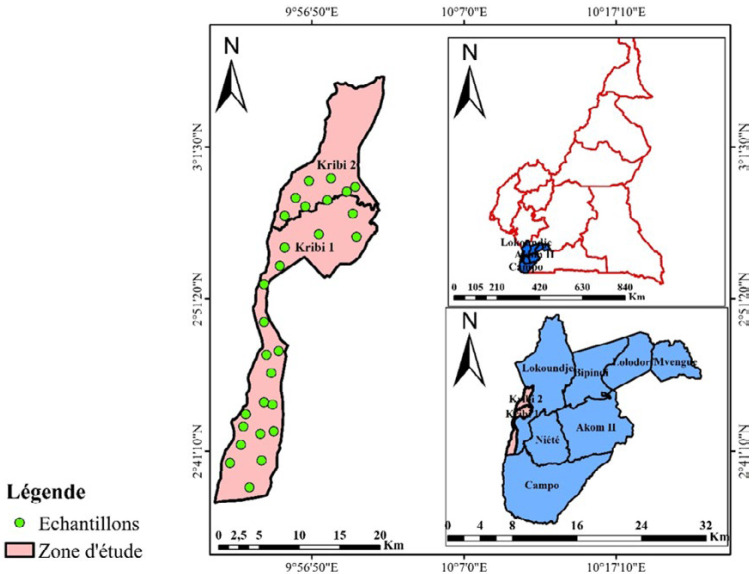
Localisation de sites d’échantillonnage à Kribi 1 (rural) et 2 (urbain)

Cette étude a été menée dans la commune de Kribi située dans le golfe de Guinée en bordure de l’Océan Atlantique (Fig. 1). Le climat est tropical humide, de type équatorial, caractérisé par quatre saisons. La température moyenne annuelle est comprise entre 27 °C et 37 °C et les précipitations moyennes annuelles sont de l’ordre de 2 970 mm. Le paludisme y est endémique et à recrudescence saisonnière. La végétation est en continuité de la forêt congolaise. Le réseau hydrographique, dense, est constitué des rivières Nyong, Ntem, Lokoundje, Lobé, Kienké, etc. La Kienké qui traverse le site d’étude est considérée comme un gîte permanent d’anophèles. Les populations vivent principalement de la pêche. Cependant, l’élevage, la cueillette, le commerce et l’agriculture y sont également pratiqués. L’usage de pesticides en agriculture est fréquent [12,20]. L’étude a eu lieu dans deux sites différents au plan écologique : Kribi rural et Kribi urbain.

Kribi rural (02°55’N, 09°55’E) est un arrondissement s’étendant sur 334 km^2^ pour une population de 22 681 habitants. Cet arrondissement est en cours d’anthropisation du fait de sa proximité avec le port en eau profonde. Plusieurs édifices sont en construction à travers le site donnant lieu à la destruction du couvert végétal. Les routes, non bitumées pour la plupart et parsemées de nids de poule, ainsi que la présence d’une grande décharge d’ordures, constituent des facteurs de prolifération des moustiques surtout en saison de pluies. Les moyens de protection sont principalement les moustiquaires [20].

Kribi urbain (02°57’N, 09°55^’^E), est un arrondissement qui s’étend sur 125 km^2^ pour une population estimée à environ 40 000 habitants. Les habitations sont majoritairement en matériaux provisoires. Les MILDA y constituent un des outils de protection contre les moustiques.

La durée de vie d’une moustiquaire se réfère à la période pendant laquelle elle remplit efficacement sa fonction de protection contre les moustiques, notamment en les empêchant d’y pénétrer et, si elle est traitée par un insecticide, de maintenir un effet répulsif [27].

L'Organisation mondiale de la Santé (OMS) définit la durée de vie fonctionnelle des MILDAcomme une utilisation effective pendant au moins 3 ans et une capacité à résister à au moins 20 lavages standard sans perdre significativement l’activité insecticide [27].

Plusieurs critères permettent de considérer une moustiquaire « hors service ». Il s’agit, par exemple, de la perte d’efficacité insecticide (lorsque l’insecticide ne tue plus ou ne repousse plus les moustiques à un niveau satisfaisant). Ce critère est évalué grâce aux tests standards (tests en cônes) pour lesquels l’efficacité doit rester supérieure à 80 % de mortalité des moustiques après contact [14]. En cas de dommages physiques (trous, déchirures, mailles effilochées), les moustiques peuvent entrer dans la moustiquaire et compromettre sa protection, même si l’insecticide est encore présent [8]. Même si une moustiquaire est intacte, on considère souvent qu’elle arrive en fin de vie après 2 à 3 ans d’utilisation dans des conditions réelles, même si l’OMS recommande 3 ans minimum [14]. Selon les lignes directrices de l’OMS et le cadre d’évaluation scientifique, les principaux critères de préqualification d’une moustiquaire s’appuient sur les recommandations officielles de l’organisme, notamment sur l’évaluation des moustiquaires imprégnées d’insecticide et les standards de test de performance développés par le *Pesticide Evaluation Scheme*, et le programme de préqualification des produits de lutte antivectorielle [29]. La moustiquaire doit être fabriquée selon des normes de qualité strictes : la matière doit être en polyester ou polyéthylène avec traitement insecticide intégré ou appliqué en usine, d’intégrité physique durable, de tissu résistant, sans défauts majeurs de fabrication et selon un système de management qualité accrédité (ISO 9001 ou équivalent) pour toutes les étapes de fabrication.

En 2019, les ménages des quartiers les plus précaires, présentant une grande promiscuité et une population élevée, ont été choisis en priorité. Après obtention du consentement éclairé des chefs de ménage des sites d’étude, les moustiquaires en cours d’utilisation ont été collectées en prélude à une distribution de masse. Il a été demandé à ces interlocuteurs de remplir un questionnaire sur la provenance, la date et le mode d’acquisition, l’utilisation et l’entretien (nombre et matériel de lavage) des MILDA. Le niveau d’utilisation était jugé régulier si le ménage utilisait sa moustiquaire toutes les nuits de la semaine précédant l’enquête (taux d’utilisation), irrégulier si le ménage ne l’utilisait que quelques nuits, et nul si la moustiquaire n’était pas utilisée durant toute la semaine. Il a de même été déterminé le nombre de moustiquaires distribué ou utilisé pour le nombre total d’espaces de couchage (taux de couverture).

Chaque MILDA échantillonnée a été remplacée par une nouvelle, puis identifiée par un code formé à partir de la marque, de la localité et du nom du chef de ménage, rangée dans un sac en plastique et conservée à 4 °C pour une évaluation ultérieure en laboratoire. Les étiquettes permanentes fixées à la moustiquaire, d’une part, et les dépliants illustrés placés dans le sac ou emballage en plastique contenant la moustiquaire (et indiquant comment traiter celle-ci et en prendre soin), d’autre part, ont été considérés. Les étiquettes fixées sur les moustiquaires renseignaient sur:

- le type de fibre;
- les dimensions en cm (largeur, longueur et hauteur pour les moustiquaires rectangulaires), et hauteur pour les moustiquaires coniques;
- les instructions de lavage (par exemple, utiliser du savon mais ni chlore ni eau de Javel);
- une instruction précisant de ne pas laisser la moustiquaire au soleil;
- un avertissement concernant l’effet du lavage qui élimine peu à peu l’insecticide.

Dans les cas où l’étiquette n’était plus présente, nous demandions au propriétaire à quoi ressemblait cette étiquette ou l’emballage de la moustiquaire. Pour ce faire, nous utilisions des fiches supports présentant des photos de toutes les MILDA couramment utilisées.

Le nombre, la taille et l’emplacement des déchirures ont été déterminés pour chaque moustiquaire. Quatre catégories de trous (0,5 cm ≤ taille 1 ≤ 2,5 cm; 2,5 cm < taille 2 ≤ 10 cm; 10 cm < taille 3 ≤ 25 cm; taille 4 ˃ 25 cm) précédemment définies par Kilian *et al*. [15] ont été retenues. L’indice proportionné des trous (pHI) a été calculé pour chaque moustiquaire inspectée afin d’évaluer son intégrité physique selon la formule:

pHI = (n tr1) + (n tr2 x 23) + (n tr3 x 196) + (n tr4 x 578)

où n tr = nombre de trous [27]. Le pHI a permis de catégoriser les échantillons en 3 types selon les dommages observés:

- état physique bon : pHI compris entre 0 et 64;
- état physique acceptable : pHI compris entre 65 et 642;
- état physique dégradé : pHI de 643 ou plus.

L’efficacité biologique des moustiquaires a été évaluée par des tests utilisant des cônes [27]. La collecte des larves d’anophèles a eu lieu dans les quartiers de Kribi rural et de Kribi urbain de février à juin 2019. Toutes les collections d’eau susceptibles de contenir les larves d’anophèles ont été visitées en utilisant la méthode de « *dipping* ». Les stades pré-imaginaux obtenus ont été mis en élevage [6]. Les adultes obtenus ont été identifiés à l’aide des clés de Gillies & De Meillon et de Gillies & Coetzee [9,10] puis conservés dans des cages pour les tests de bio-efficacité. La souche Kisumu d’*An. gambiae* originaire du Kenya et provenant de l’OCEAC, mise en élevage dans les mêmes conditions que les souches naturelles, a servi de témoin.

Cinq échantillons de tulle, d’une dimension de 25 cm x 25 cm chacun, ont été prélevés au hasard sur les 4 faces et sur le toit de 5 moustiquaires testées pour Kribi rural et 5 pour Kribi urbain, soit un total de 50 échantillons de tulle testés. Dix moustiques femelles d’*An. gambiae* s.l. âgées de 2 à 5 jours ont été exposées pendant 3 min à chacun des 5 échantillons de chaque moustiquaire, soit 500 anophèles testés. Le même effectif de moustiques a été exposé à un tulle de moustiquaire non imprégné d’insecticide pour servir de contrôle négatif par jour de test, soit 500 spécimens au total. Après 3 min d’exposition, les moustiques étaient transférés dans des gobelets plastiques recouverts de tulle non imprégné d’insecticide, nourris avec une solution sucrée à 10 % et maintenus à une température de 27 ± 3 °C et à une humidité relative de 75 ± 10 %. Le nombre de moustiques assommés ou *knock-down* (KD) a été relevé toutes les 10 min pendant une heure. L’ensemble des gobelets a été gardé en observation pendant 24 h au terme desquelles la mortalité a été enregistrée. La souche Kisumu, totalement sensible aux pyréthrinoïdes, a été utilisée comme souche de référence afin de vérifier l’efficacité intrinsèque de l’insecticide imprégné dans les MILDA.

Le test a été interprété selon les critères de l’OMS [26] pour lesquels une MILDA est considérée efficace si elle entraîne un taux de KD supérieur ou égal à 95 % et/ou une mortalité supérieure ou égale à 80 % respectivement en une heure et 24 h. L’efficacité minimale est ≥ 50 % de mortalité ou ≥ 75 % de KD et l’absence d’efficacité < 50 % de mortalité ou < 75 % de KD.

Les données ont été saisies dans un tableur Excel et analysées à l’aide du logiciel *Statistical Package for Social Sciences* (SPSS) version 22.0. Le test H de Kruskall-Wallis a été utilisé pour comparer les différences de taille entre les trous, le test R de Pearson pour observer les corrélations entre la fréquence de lavage de MILDA et les pHI, et le test de chi² pour comparer le taux de mortalité des souches au seuil de significativité de 5 %.

## Résultats

Au total, 540 ménages dont 235 à Kribi urbain et 305 à Kribi rural ont été enquêtés permettant le recensement de 1 260 moustiquaires (546 à Kribi urbain et 714 à Kribi rural). En milieu urbain, 17,9 % de ménages (n = 42) possédaient au moins une moustiquaire pour 2 personnes et 16,4 % (n = 50) en milieu rural (Tableau I). Huit marques de moustiquaires étaient relevées, les plus répandues étant Interceptor (290/448) et PermaNet 2.0 (114/448) (Tableau IIa).

**Tableau I:** Paramètres généraux relatifs à la couverture et à l’utilisation des MILDA à Kribi en 2019

Modalités	Kribi urbain	Kribi rural	Total
Nombre de ménages enquêtés	235	305	540
Nombre de personnes enquêtées	1 343	1 750	3 093
Nombre d'enfants de moins de 5 ans	275 (20,5%)	46 (26,3%)	735 (23,8%)
nombre d'enfants (-5 ans) utilisant une MILDA	242 (88%)	409 (88,9%)	651 (88,6%)
nombre de femmes enceintes utilisant une MILDA	4/6	1/3	5/9
total des espaces de couchage	631	761	1 392
utilisation des MILDA par les ménages	210 (89,4%)	248 (81,3%)	458 (84,8%)
MILDA disponibles	546	714	1 260
MILDA en utilisation régulière	433	525	958
nombre de lavages	3 597	3 646	7 243
usage de détergents	124	52	176
moyenne de MILDA par ménage	2,34 ± 0,127	2,31 ± 0,128	2,33 ± 0,091
ménages avec au moins une MILDA	211 (89,8%)	245 (80,3%)	456 (84,4%)
ménages avec au moins une MILDA pour 2 personnes	42 (19,9%)	50 (20,4%)	92 (20,2%)
taux de couverture (%)	86,5	93,8	90,2
taux d'utilisation régulière (%)	79,3	73,5	76,4

Le taux d’utilisation régulière des moustiquaires était comparable en milieu urbain (79,3 %) et rural (73,5 %). Le nombre d’enfants de moins de 5 ans utilisant une moustiquaire était de 651 (88,6 %). Parmi les 9 femmes enceintes, 5 dormaient sous une moustiquaire.

Les taux de couverture en MILDA étaient de 86,5 % et de 93,8 % respectivement en milieux urbain et rural (Tableau I).

Interceptor était la marque la plus répandue (161/241 en zone rurale et 128/207 en milieu urbain), suivie de PermaNet, Olyset Net, Yorkool (Tableau II).

**Tableau II:** Composition, marque et recommandations recueillies sur les MILDA en condition opérationnelle

Marques de moustiquaires	Insecticide imprégné (mg/m²)	Type de fibres /résistance*	Kribi rural	Kribi urbain	Total (%)	Taille (pouce²)
Interceptor	Alpha-cyperméthrine (200)	Polyester 75, 100	161	128	289 (64,5%)	156-177
PermaNet 2.0	Deltaméthrine (55)	Polyester 75, 100	54	60	114 (25,4%)	156-177
Olyset Net	Perméthrine (1000)	Polyéthylène 150	9	12	21 (4,6%)	75
Yorkool	Deltaméthrine (55)	Polyester 75, 100	13	4	17 (3,8%)	156-177
Royal Sentry	Alpha-cyperméthrine (260)	Polyéthylène 145	-	1	1 (0,2%)	132
Netto	Deltaméthrine (80)	Polyester 75, 100, 150	2	-	2 (0,4%)	156-177
DuraNet	Alpha-cyperméthrine (260)	Polyéthylène 145	1	1	2 (0,4%)	132
Panda Net	Deltaméthrine (63)	Polyéthylène 100, 115	1	1	2 (0,4%)	136 & 200

* exprimée en deniers

Les moustiquaires à fibre en polyester étaient les plus présentes en zone rurale et urbaine (95,9 % et 91,8 % respectivement), sans différence significative. Les moustiquaires en polyéthylène étaient moins utilisées : 11/241 et 15/207 en zones rurale et urbaine, sans différence significative.

Un total de 2 646 trous a été trouvé sur un sous échantillon de 122 moustiquaires, dont 1 393 (52,6 %) de taille I, 718 (27,1 %) de taille II, 384 (14,3 %) de taille III et 151 (5,7 %) de taille IV (déchirures) Le nombre moyen de trous par moustiquaire était de 22,32 ± 0,32 à Kribi rural et de 21,03 ± 0,33 à Kribi urbain (Tableau III).

**Tableau III:** Moyennes de type de trous obtenues à Kribi rural et Kribi urbain pour chaque type de moustiquaire

Sites	Kribi urbain	Kribi rural	Total (%)
Nombre de MILDA testées	62	60	122
Nombre de trous	1 384	1 262	2 646
Type I	n = 738 m = 11,90 ± 0,57	n = 655 m = 10,92 ± 0,76	n = 1 393 m = 11,42 ± 0,47
Type II	n = 376 m = 6,06 ± 0,45	n = 342 m = 5,70 ± 0,42	n = 718 m = 5,88 ± 0,31
Type III	n = 188 m = 3,03 ± 0,26	n = 196 m = 3,27 ± 0,32	n = 384 m = 3,15 ± 0,29
Type IV	n = 82 m = 1,32 ± 0,13	n = 69 m = 1,15 ± 0,12	n = 151 m = 1,23 ± 0,13

L’analyse des pHI a montré que les moustiquaires de la marque PermaNet (1 435 trous ± 117) étaient plus dégradées que les autres marques à Kribi rural alors que les moustiquaires de marques Yorkool (2 004 trous ± 328) l’étaient davantage à Kribi urbain (Tableau IV).

**Tableau IV:** Indices de trous (pHI) en fonction des marques de moustiquaires

Marques	Nombre de MILDA	pHI - État physique	Nombre de MILDA	pHI - État physique
Interceptor	161	786 ± 63 dégradé	128	1 095 ± 60 dégradé
Olyset Net	9	1 182 ± 219 dégradé	12	1 836 ± 227 dégradé
PermaNet	54	1 435 ± 117 dégradé	60	1 527 ± 117 dégradé
Yorkool	13	1 100 ± 206 dégradé	4	2 004 ± 328 dégradé
Royal sentry	0	0	1	1 103 dégradé
Netto	2	1 034 dégradé	0	0
DuraNet	1	791	1	438 acceptable
Panda Net	1	0 bon	1	0 bon
**Total**	**241**	**791 ± 75 dégradé**	**207**	**1 333 ± 234 dégradé**

Le nombre de MILDA ayant subi entre 10 et 15 lavages après 36 mois d’utilisation était plus élevé à Kribi rural et celui de MILDA ayant subi plus de 20 lavages l’était à Kribi urbain (Tableau V). Une corrélation positive et significative entre la fréquence de lavage et les pHI aussi bien à Kribi rural (R = 0,612, p ≤ 10^-4^) qu’à Kribi urbain (R = 0,600, p ≤ 10^-4^) a été observée.

**Tableau V:** Modalités d’entretien des MILDA (fréquence de lavage, type de savon, mode de séchage et durée de vie)

		Kribi urbain		Kribi rural	
	Modalités d’entretien	Nombre de MILDA	pHI - État physique	Nombre de MILDA	pHI - État physique
**Méthodes d'entretien**	**Fréquence de lavages**	**241**		**207**	
	[0-5[	1	420 acceptable	2	2 232 ± 2 224 dégradé
	[5-10[	26	591 ± 126 (dégradé)	30	1 006 ± 97 dégradé
	[10-15[	106	610 ± 69 acceptable	55	713 ± 61 dégradé
	[15-20[	45	1 193 ± 123 dégradé	16	1 316 ± 153 dégradé
	≥20	63	1 519 ± 97 dégradé	103	1 636 ± 76 dégradé
	**Type de savon**	**241**		**207**	
	savon ordinaire	190	624 ± 39	83	557 ± 35
	détergent corrosif	51	1 830 ± 84	124	1 754 ± 57
	**Mode de séchage**	**114**		**100**	
	soleil	52	2 258 ± 61	68	1 871 ± 84
	ombre	62	93 ± 11	32	225 ± 32
**Période de validité (en mois)**	**Durée de vie (mois)**	**235**		**205**	
	[0-10[	6	1 134 ± 433	12	689 ± 99
	[10-20[	34	826 ± 121	58	967 ± 76
	[20-30[	82	493 ± 61	26	844 ± 105
	[30-40[	94	1 246 ± 87	93	1 528 ± 87
	40≤	17	1 528 ± 233	16	2 037 ± 178
	Test R		0,612		0,600
**Types de fibres**	Polyester	231	958 ± 54	191	1 264 ± 57
	Polyéthylène	10	967 ± 221	15	1 468 ± 234

L’utilisation des savons ordinaires était plus fréquente à Kribi rural (190/241) qu’à Kribi urbain (83/207) tandis que l’usage de détergents corrosifs était plus marqué à Kribi urbain (124/207) qu’à Kribi rural (51/241). Le lavage avec le savon ordinaire est associé à des pHI moins élevés que le lavage aux détergents corrosifs aussi bien à Kribi rural (p ≤ 10^-4^) qu’à Kribi urbain (p ≤ 10^-4^) (Tableau V).

Le séchage des MILDA au soleil après lavage était une pratique très répandue à Kribi urbain (68 ménages/100) et le séchage à l’ombre était plus fréquent en zone rurale (62 ménages/114). Les pHI étaient significativement plus élevés pour les MILDA étalées au soleil après lavage que pour les MILDA étalées à l’ombre aussi bien à Kribi rural (p = 0,001) qu’à Kribi urbain (p = 0,042). Il n’existe pas de corrélation entre la durée de vie des MILDA (liée à un certain nombre de facteurs notamment les méthodes d’entretien, le temps d’utilisation, le type de fibres, les recommandations du fabricant, etc.) et les pHI dans les deux sites d’étude (Tableau V).

Les taux de mortalité baissent avec l’augmentation des fréquences de lavage des MILDA des marques Olyset Net, PermaNet et Interceptor aussi bien à Kribi rural qu’à Kribi urbain (Tableau VI).

**Tableau VI:** Taux de mortalité d’*Anopheles gambiae* (souche locale) et de la souche Kisumu 24 h après exposition à 10 MILDA

	Kribi rural					Kribi urbain	Kisumu	
Insecticides	Nombre de MILDA	Souche locale (mortalité %)	Souche Kisumu (mortalité %)	Marque	Nombre de MILDA	Souche locale (mortalité %)	Souche Kisumu (mortalité %)	Marque
**Perméthrine**	**1**			Olyset Net	**2**			Olyset Net
[0-10 lavages]	1	2 %	55 %		1	50 %	96 %	
[10-20 lavages]	0				1	41 %	99 %	
**Deltaméthrine**	**2**			PermaNet rectangulaire	**1**			PermaNet rectangulaire
[0-10 lavages]	1	60 %	95 %		0			
[10-20 lavages]	1	45 %	98 %		1	4 %	90 %	
**Alpha-cyperméthrine**	**2**			Interceptor	**2**			Interceptor
[0-10 lavages]	1	1 %	22 %		1	1 %	45 %	
[10-20 lavages]	1	1 %	42 %		1	33 %	79 %	
**Total**	**5**				**5**			

## Discussion

La technologie des MILDA a été développée pour remédier au faible taux d’imprégnation des moustiquaires conventionnellement traitées [24]. Leur distribution et leur utilisation sont devenues une priorité pour les programmes nationaux de lutte contre le paludisme [1,3]. Cependant, la promotion des moustiquaires ne peut être effective que si elle s’inscrit dans un ensemble de mesures visant à améliorer la qualité de la vie des populations. À Kribi, les taux d’utilisation des MILDA étaient de 73 % en zone rurale et 79 % en milieu urbain. Ce taux élevé d’utilisation peut être associé à la forte densité culicidienne observée dans la zone d’étude [16]. Nos résultats sont en cohérence avec ceux rapportés au Bénin et au Mozambique [22,23]. Selon ces auteurs ce taux était de 25 % à Nampula et > 60 % au Bénin. Bien que l’utilisation des MILDA soit élevée lorsque celles-ci sont disponibles, la couverture réelle reste inférieure au seuil de couverture recommandé par l’OMS (≥80 %). Une large couverture d’une population (> 80 %) en moustiquaires imprégnées occasionnerait la protection même de ceux qui ne les utilisent pas [24]. Elle permettrait une réduction de la transmission et du taux d’incidence des fièvres palustres comme l’a montré l’introduction de masse de moustiquaires imprégnées de lambda-cyhalothrine en zone de savane septentrionale de Côte d’Ivoire [15].

Pour assurer avec efficacité son rôle de protection, une moustiquaire doit constituer une barrière physique infranchissable par les moustiques. L’usure du temps, le mode d’entretien (fréquence de lavage, type de savon utilisé, mode de séchage) sont autant de facteurs pouvant impacter l’intégrité physique d’une MILDA. Notre étude a montré une corrélation positive entre les pHI et la fréquence de lavages à Kribi rural. Ces résultats sont conformes à ceux enregistrés dans des études menées dans le centre et le sud du Cameroun [4,7,25]. Les tensions exercées sur les fibres des moustiquaires avec la fréquence élevée des lavages entraînent l’apparition des trous de type I qui vont s’agrandir en taille II, III, voire IV. Des observations similaires ont été faites dans des quartiers de la ville de Douala [19]. La nature du détergent utilisé lors des lavages peut également être à l’origine de la détérioration d’une moustiquaire. Les pHI étaient moins élevés pour les MILDA lavées au savon ordinaire que celles lavées avec les détergents corrosifs aussi bien en milieu rural qu’urbain. Les MILDA sales sont trempées dans l’eau contenant le détergent pendant plusieurs minutes voire des heures avant d’être lavées. Ce processus de lavage occasionnerait des interactions chimiques entre les molécules constitutives du détergent d’une part et le polyester ou polyéthylène d’autre part. Avec le savon en morceaux, un tel processus de trempage n’a pas lieu et les MILDA sont directement lavées. Les moustiquaires les plus utilisées étaient en fibre de polyester. Le polyester comporte de nombreux avantages par rapport au coton, au nylon et au polyéthylène : c’est une fibre plus durable qui assure une meilleure ventilation et une meilleure biodisponibilité de l’insecticide. Le polyester coûte généralement moins cher, il est plus léger, permettant de réduire les coûts de transport, il bénéficie d’un meilleur contrôle de qualité et il est plus populaire. Des campagnes de sensibilisation devraient être organisées par des agents de santé communautaire pour apprendre aux populations l’entretien des MILDA.

Les tests insecticides montrent une perte graduelle d’efficacité avec la fréquence de lavage. La marque Olyset Net est un type nouveau de moustiquaire pour laquelle l’insecticide (perméthrine utilisée à la concentration de 2 % poids/poids, soit environ 900 mg/m^2^) est incorporé par fusion dans une fibre composée de résine de polyéthylène. Ces moustiquaires auraient une durée d’efficacité de 3 ans et pourraient la conserver après 30 lavages [5]. Cependant, lavées avec un détergent corrosif, une perte de l’efficacité de l’insecticide est observée. La perméthrine et la deltaméthrine sont instables en milieu basique. À pH 9, leur durée de vie se réduit considérablement tandis qu’en milieu acide, elles sont stables. Le lavage de la moustiquaire avec de l’eau pourvue de vinaigre est à promouvoir. Pour les moustiquaires PermaNet, l’insecticide, (deltaméthrine dosée à 50 mg/m^2^) est mélangé à une résine qui enrobe les fibres en polyester. Le pyréthrinoïde ainsi fixé sur le support est progressivement relâché par la résine. En laboratoire, après 20 lavages, un contact de 3 min des moustiques avec la tulle traitée induisait toujours 100 % d’effet KD et 50 % de mortalité [6]. La baisse de l’efficacité observée *in situ* au bout de 20 lavages des moustiquaires est liée aux produits corrosifs et à leur exposition au soleil après lavage [24].

## Conclusion

La population rurale et urbaine de Kribi utilisait largement les moustiquaires en 2019, au moins pour les enfants. Le taux de couverture global par des moustiquaires était cependant élevé, entre 87 % et 94 % selon le nombre de dormeurs par moustiquaire, supérieur au taux recommandé de 80 % au moins. De même, le ratio nombre moyen de MILDA/ménage était faible 2,33 ± 0,091. Notre étude montre que la composition de la fibre, la fréquence de lavage, les détergents utilisés et le lieu de séchage (ombre ou soleil) sont des facteurs susceptibles d’agir sur l’intégrité physique et l’efficacité biologique d’une MILDA. Il n’est pas facile de standardiser les procédures de lavage et de séchage. La bio-efficacité des MILDA sur la souche locale d’*An. gambiae* s.l*.* était fortement réduite, particulièrement après des lavages répétés, suggérant un impact possible de la résistance aux pyréthrinoïdes et des pratiques d’entretien inadaptées. Une sensibilisation sur l’entretien, la bonne gestion et le remplacement peut permettre une adoption des bonnes pratiques pouvant contribuer à augmenter la durée de l’intégrité physique et de l’efficacité des MILDA.

## Consentement éclairé et considérations éthiques

L’approbation éthique de cette étude a été obtenue auprès de la Faculté des sciences de l’Université de Douala (UD). L’autorisation administrative a été obtenue conjointement auprès des sous-préfets des 2 arrondissements et du préfet du département de l’Océan (Sud-Cameroun) sous le numéro 497/ AR/L11/A1. Tous les participants à l’étude ont donné leur consentement éclairé.

## Remerciements

Nous tenons à remercier toute la population du département de l’Océan pour sa collaboration.

## Financement

Les auteurs déclarent n’avoir reçu aucun financement d’un organisme quel qu’il soit pour la réalisation de ce travail autre que leur participation propre.

## Disponibilité des données et du matériel

Les ensembles de données utilisés et/ou analysés au cours de la présente étude sont disponibles auprès de l’auteur correspondant sur demande raisonnable.

## Contributions des auteurs et autrices

RSM, PAN, JAMM et PAA ont conçu l’étude. RSM, MLOE, PMN, OENH, FNNT, RN ont effectué le travail de terrain. RSM, MLOE, PMN, MN, RN et FNNT ont réalisé les analyses de laboratoire. RSM, PAA, FNNT, WDT et MN ont fait l’analyse des données. JAMM, PAA et PAN ont supervisé l’étude. RSM, PAN, PAA et AN ont rédigé le projet original. RSM, PNA, JAMM, AN, PAA, WDT et EWE ont revu et édité la version finale. Tous les auteurs ont lu et approuvé le manuscrit final.

## Déclaration de liens d’intérêt

Aucun lien d’intérêt n’a été déclaré.
