# Supplementary material for: Use and effectiveness of long-lasting insecticide-treated nets (LLINs) in southern Cameroon
Source: Med Trop Sante Int. 2026 Apr 8;6(2):mtsi.v6i2.2026.838. [Article in French] doi: 10.48327/mtsi.v6i2.2026.838 (PMC13423292; doi:10.48327/mtsi.v6i2.2026.838)
Supplement: Related texts

# Research article: Use and effectiveness of long-lasting insecticide-treated nets (LLINs) in southern Cameroon

## Introduction

Malaria is one of the deadliest parasitic diseases in the world. Nearly 228 million people contracted malaria in 2018, resulting in approximately 409,000 reported deaths [28]. Sub-Saharan Africa is the most vulnerable region. In Cameroon, malaria is the leading cause of morbidity and mortality and constitutes a major public health problem. In 2019, the disease resulted in approximately 11,000 reported deaths and negatively impacted the economies of households and the nation as a whole [21]. The most vulnerable groups are pregnant women and children under five years of age [22]. The Cameroonian government’s National Malaria Control Program (PNLP) has defined several strategic priorities, primarily prevention through the use of insecticide-treated bed nets [21].

Using mosquito nets to ward off mosquitoes and reduce the transmission of infectious agents is an ancient practice [17]. Early trials conducted in various countries demonstrated the impact of insecticide-treated nets on malaria incidence [17]. However, these nets had the drawback of requiring regular re-treatment, at least once a year [18]. Long-lasting insecticidal nets (LLINs) were developed as an alternative. LLINs are nets that have been pre-treated in an industrial process that allows them to remain effective after at least 20 washes and retain their insecticidal properties for three to five years under normal use [26]. An insecticide from the pyrethroid family is incorporated or coated into polyester or polyethylene fibers during manufacturing and slowly released to migrate to the surface of the net, producing a deterrent, repellent, or excito-repellent effect. At high coverage levels, the benefits of using LLINs at the community level have been demonstrated. Beyond personal protection, a mass effect on the vector population is observed.

LLINs are one of the cornerstones of vector control (VC). In sub-Saharan Africa, for example, the proportion of the population with access to an LLIN increased from less than 2% in 2000 to 67% in 2015. Some countries have achieved universal coverage [26]. However, the key question of their actual lifespan, in terms of physical integrity and bio-efficacy, remains unanswered. There is a lack of research on the functional lifespan and performance variation of different LLINs under various usage conditions. Studies conducted in Uganda revealed that 45 to 78% of nets were damaged within one year of use [13]. In Kenya and Benin, a faster-than-expected decline in bioefficacy was noted, raising questions about their effective lifespan [2-6,11]. A study in Laos found that approximately 40% of the nets were damaged after two to three years of use [30].

The town of Kribi in southern Cameroon was among those that benefited from a mass distribution of LLINs in 2019. This study assesses the physical integrity and bioefficacy of LLINs after three years of household use in Kribi.

## Materials and methods

This study was conducted in the municipality of Kribi, which is located on the Atlantic coast of the Gulf of Guinea (Fig. 1). The climate is humid tropical, of the equatorial type, and is characterized by four seasons. The average annual temperature ranges from 27°C to 37°C, and the average annual precipitation is approximately 2,970 mm. Malaria is endemic there and exhibits seasonal resurgence. The vegetation is an extension of the Congolese forest. The dense river network consists of the Nyong, Ntem, Lokoundje, Lobé, and Kienké rivers, among others. The Kienké River, which flows through the study site, is a known breeding ground for *Anopheles* mosquitoes. The local population primarily relies on fishing for sustenance. However, they also engage in livestock raising, foraging, trade, and agriculture. The use of pesticides in agriculture is common [12,20]. The study was conducted at two ecologically distinct sites: rural and urban Kribi.

**Figure 1:** Location of sampling sites in Kribi 1 (rural) and 2 (urban)

The rural district of Kribi (02°55’N, 09°55’E) covers 334 km² and has a population of 22,681. Due to its proximity to the deep-water port, this district is undergoing rapid urbanization. Several buildings are under construction throughout the area, which has led to the destruction of vegetation. The unpaved roads riddled with potholes and the large garbage dump contribute to mosquito proliferation, especially during the rainy season. Mosquito nets are the primary means of protection [20].

Urban Kribi (02°57’N, 09°55’E) is a district spanning 125 km² with an estimated population of 40,000. Most dwellings are made of temporary materials. LLINs are one of the tools used for protection against mosquitoes.

A mosquito net’s lifespan refers to the period during which it effectively fulfills its function of protecting against mosquitoes by preventing them from entering, and if treated with an insecticide, by maintaining a repellent effect [27].

The World Health Organization (WHO) defines the functional lifespan of LLINs as effective use for at least three years and the ability to withstand at least 20 standard washes without losing significant insecticidal activity [27].

There are several criteria that determine when a mosquito net is considered “out of service.” These include loss of insecticidal efficacy, or when the insecticide no longer kills or repels mosquitoes adequately. This criterion is assessed using standard cone tests, in which efficacy must result in at least 80% mosquito mortality upon contact [14]. Physical damage, such as holes, tears, or frayed mesh, can allow mosquitoes to enter the net and compromise its protective function, even if the insecticide is still present [8]. A net is often considered to have reached the end of its useful life after two to three years of use under real-world conditions, even if it is intact. However, the WHO recommends a minimum of three years [14].

According to WHO guidelines and the scientific evaluation framework, the main criteria for prequalifying a mosquito net are based on the organization’s official recommendations. These recommendations focus particularly on evaluating insecticide-treated nets and on developing performance testing standards for the Pesticide Evaluation Scheme and the Vector Control Product Prequalification Program [29]. Mosquito nets must be manufactured according to strict quality standards. They must be made of polyester or polyethylene with an integrated or factory-applied insecticide treatment. They must also be made of durable, resistant fabric and be free of major manufacturing defects. All stages of manufacturing must be produced under an accredited quality management system (ISO 9001 or equivalent).

In 2019, households in the most vulnerable neighborhoods, characterized by high population density and overcrowding, were prioritized. After obtaining informed consent from the heads of households at the study sites, the nets currently in use were collected prior to mass distribution. Participants were asked to complete a questionnaire regarding the source, date, and method of acquisition of LLINs, as well as how they were used and maintained (frequency and cleaning materials). Use was considered regular if the household used its mosquito net every night during the week before the survey, irregular if it was used only a few nights, and nonexistent if it was not used at all during that week. The number of nets distributed or used relative to the total number of sleeping spaces was also determined (coverage rate).

Each LLIN sampled was replaced with a new one, identified by a code based on the brand, location, and name of the head of household, placed in a plastic bag, and stored at 4°C for subsequent laboratory evaluation. We considered the permanent labels attached to the mosquito net and the illustrated leaflets placed in the plastic bag or packaging containing the net, which indicate how to treat and care for it.

The labels on the nets provided information on:

- the type of fiber;
- dimensions in centimeters (width, length, and height for rectangular nets and height for conical nets);
- washing instructions (e.g., use soap, but not chlorine or bleach);
- an instruction not to leave the mosquito net in the sun;
- a warning about the gradual removal of the insecticide through washing.

When the label was missing, we asked the owner what it looked like or what the packaging looked like. We used reference sheets displaying photos of all commonly used LLINs to do this.

We determined the number, size, and location of tears for each mosquito net. We used four categories of holes previously defined by Kilian *et al.* [15]: 0.5 cm ≤ size 1 ≤ 2.5 cm; 2.5 cm < size 2 ≤ 10 cm; 10 cm < size 3 ≤ 25 cm; and size 4 > 25 cm. We calculated the Proportional Hole Index (pHI) for each inspected mosquito net to assess its physical integrity using the formula:

pHI = (n tr1) + (n tr2 x 23) + (n tr3 x 196) + (n tr4 x 578)

where n tr is the number of holes [27]. The pHI categorized the samples into three types based on the observed damage:

- good physical condition: pHI between 0 and 64;
- acceptable physical condition: pHI between 65 and 642;
- deteriorated physical condition: pHI of 643 or higher.

The biological efficacy of the mosquito nets was assessed using cone tests [27]. *Anopheles* larvae were collected in rural and urban districts in Kribi from February to June of 2019. All water sources likely to contain Anopheles larvae were surveyed using the “dipping” method. The pre-imago stages obtained were reared [6]. The resulting adults were identified using Gillies & De Meillon’s and Gillies & Coetzee’s [9,10] keys and then kept in cages for bioefficacy testing. The Kisumu strain of *An. gambiae*, native to Kenya and obtained from OCEAC, was reared under the same conditions as the natural strains and served as a control.

Five 25 cm x 25 cm mesh samples were randomly selected from the four sides and top of five mosquito nets tested in rural and urban Kribi, for a total of 50 net samples. Ten 2- to 5-day-old female *An. gambiae* s.l. mosquitoes were exposed to each of the five samples from each net for three minutes, for a total of 500 mosquitoes tested. The same number of mosquitoes was exposed to a mosquito net fabric not impregnated with insecticide to serve as a negative control on each test day, for a total of 500 specimens. After three minutes, the mosquitoes were transferred to plastic cups lined with untreated netting. They were fed a 10% sugar solution and maintained at a temperature of 27 ± 3°C and a relative humidity of 75 ± 10%. The number of stunned or knocked-down (KD) mosquitoes was recorded every 10 minutes for one hour. All cups were kept under observation for 24 hours, after which mortality was recorded. The Kisumu strain, fully susceptible to pyrethroids, served as a reference strain to verify the intrinsic efficacy of the insecticide impregnated in the LLINs.

The test was interpreted according to WHO criteria [26], under which a LLIN response is considered effective if it results in a KD rate of 95% or higher and/or a mortality rate of 80% or higher within one hour and 24 hours, respectively. Minimum efficacy is defined as a mortality rate of at least 50% or a KD rate of at least 75%, and a lack of efficacy is defined as a mortality rate of less than 50% or a KD rate of less than 75%.

The data were entered into an Excel spreadsheet and analyzed using Statistical Package for the Social Sciences (SPSS) software, version 22.0. The Kruskal–Wallis H test was used to compare differences in hole size, the Pearson r test was used to examine correlations between LLIN washing frequency and pHI, and the chi² test was used to compare mortality rates of strains at a 5% significance level.

## Results

A total of 540 households were surveyed, including 235 in urban Kribi and 305 in rural Kribi. This resulted in the identification of 1,260 mosquito nets, 546 of which were in urban Kribi and 714 of which were in rural Kribi. In urban areas, 17.9% of households (n = 42) owned at least one net for two people, compared to 16.4% in rural areas (n = 50) (Table I). Eight brands of mosquito nets were identified; the most common were Interceptor (290/448) and PermaNet 2.0 (114/448) (Table IIa). The rate of regular mosquito net use was comparable in urban (79.3%) and rural (73.5%) areas. Of the children under five years of age, 651 (88.6%) used a mosquito net. Among the nine pregnant women, five slept under a mosquito net.

**Table I:** General parameters regarding the coverage and use of LLINs in Kribi in 2019

Modalities	Urban Kribi	Rural Kribi	Total
Number of households surveyed	235	305	540
Number of individuals surveyed	1 343	1 750	3 093
Number of children under 5 years of age	275 (20,5%)	46 (26,3%)	735 (23,8%)
Number of children (under 5) using a LLIN	242 (88%)	409 (88,9%)	651 (88,6%)
Number of pregnant women using a LLIN	4/6	1/3	5/9
Total number of sleeping areas	631	761	1 392
Household use of LLINs	210 (89,4%)	248 (81,3%)	458 (84,8%)
Available LLINs	546	714	1 260
LLINs in regular use	433	525	958
Number of washes	3 597	3 646	7 243
Use of detergents	124	52	176
Average number of LLINs per household	2,34 ± 0,127	2,31 ± 0,128	2,33 ± 0,091
Households with at least one LLIN	211 (89,8%)	245 (80,3%)	456 (84,4%)
Households with at least one LLIN for every 2 people	42 (19,9%)	50 (20,4%)	92 (20,2%)
Coverage rate (%)	86,5	93,8	90,2
Rate of regular use (%)	79,3	73,5	76,4

The LLIN coverage rate was 86.5% in urban areas and 93.8% in rural areas (Table I).

Interceptor was the most common brand, at 161/241 in rural areas and 128/207 in urban areas, followed by PermaNet, Olyset Net, and Yorkool (Table II).

**Table II:** Composition, model, and recommendations gathered from LLINs in operational conditions

Mosquito net brands	Insecticide content (mg/m²)	Fiber type / strength*	Rural Kribi	Urban Kribi	Total (%)	Size (inches²)
Interceptor	Alpha-cypermethrin (200)	Polyester 75, 100	161	128	289 (64,5%)	156-177
PermaNet 2.0	Deltamethrine (55)	Polyester 75, 100	54	60	114 (25,4%)	156-177
Olyset Net	Permethrine (1000)	Polyethylene 150	9	12	21 (4,6%)	75
Yorkool	Deltamethrine (55)	Polyester 75, 100	13	4	17 (3,8%)	156-177
Royal Sentry	Alpha-cypermethrin (260)	Polyethylene 145	-	1	1 (0,2%)	132
Netto	Deltamethrine (80)	Polyester 75, 100, 150	2	-	2 (0,4%)	156-177
DuraNet	Alpha-cypermethrin (260)	Polyethylene 145	1	1	2 (0,4%)	132
Panda Net	Deltamethrine (63)	Polyethylene 100, 115	1	1	2 (0,4%)	136 & 200

expressed in deniers

Polyester fiber nets were the most common in both rural (95.9%) and urban (91.8%) areas, with no significant difference. Polyethylene nets were used less frequently: 10/241 and 16/207 in rural and urban areas, respectively, with no significant difference.

A total of 2,646 holes were found in a subsample of 122 mosquito nets, including 1,393 (52.6%) size I, 718 (27.1%) size II, 384 (14.3%) size III, and 151 (5.7%) size IV tears. The average number of holes per net was 22.32 ± 0.32 in rural Kribi and 21.03 ± 0.33 in urban Kribi (Table III).

**Table III:** Average hole sizes recorded in rural Kribi and urban Kribi for each type of mosquito net

Locations	Urban Kribi	Rural Kribi	Total (%)
Number of LLINs tested	62	60	122
Number of holes	1 384	1 262	2 646
Type I	n = 738 m = 11,90 ± 0,57	n = 655 m = 10,92 ± 0,76	n = 1 393 m = 11,42 ± 0,47
Type II	n = 376 m = 6,06 ± 0,45	n = 342 m = 5,70 ± 0,42	n = 718 m = 5,88 ± 0,31
Type III	n = 188 m = 3,03 ± 0,26	n = 196 m = 3,27 ± 0,32	n = 384 m = 3,15 ± 0,29
Type IV	n = 82 m = 1,32 ± 0,13	n = 69 m = 1,15 ± 0,12	n = 151 m = 1,23 ± 0,13

Analysis of the pHI showed that PermaNet brand nets (1,435 holes ± 17) were more degraded than other brands in rural Kribi. In contrast, Yorkool brand nets (2,004 holes ± 328) were more degraded in urban Kribi (Table IV).

**Table IV:** Hole Index (pHI) by mosquito net brand

Brands	Number of LLINs	pHI - Physical Condition	Number of LLINs	pHI - Physical Condition
Interceptor	161	786 ± 63 degraded	128	1 095 ± 60 degraded
Olyset Net	9	1 182 ± 219 degraded	12	1 836 ± 227 degraded
PermaNet	54	1 435 ± 117 degraded	60	1 527 ± 117 degraded
Yorkool	13	1 100 ± 206 degraded	4	2 004 ± 328 degraded
Royal sentry	0	0	1	1 103 degraded
Netto	2	1 034 degraded	0	0
DuraNet	1	791	1	438 acceptable
Panda Net	1	0 bon	1	0 bon
**Total**	**241**	**791 ± 75 degraded**	**207**	**1 333 ± 234 degraded**

A higher percentage of LLINs in rural Kribi had undergone between 10 and 15 washes after 36 months of use, and a higher percentage of LLINs in urban Kribi had undergone more than 20 washes (Table V). A positive and significant correlation was observed between washing frequency and pHI in both rural (R = 0.612, p ≤ 10^-4^) and urban (R = 0.600, p ≤ 10^-4^) Kribi.

**Table V:** Care Instructions for MILDA (washing frequency, type of detergent, drying method, and lifespan)

		Urban Kribi		Rural Kribi	
	Care modalities	Number of LLINs	pHI - Physical Condition	Number of LLINs	pHI - Physical Condition
**Care Methods**	**Washing Frequency**	**241**		**207**	
	[0-5[	1	420 acceptable	2	2 232 ± 2 224 degraded
	[5-10[	26	591 ± 126 (dégradé)	30	1 006 ± 97 degraded
	[10-15[	106	610 ± 69 acceptable	55	713 ± 61 degraded
	[15-20[	45	1 193 ± 123 degraded	16	1 316 ± 153 degraded
	≥20	63	1 519 ± 97 degraded	103	1 636 ± 76 degraded
	**Type of soap**	**241**		**207**	
	regular soap	190	624 ± 39	83	557 ± 35
	corrosive detergent	51	1 830 ± 84	124	1 754 ± 57
	**Drying method**	**114**		**100**	
	sun	52	2 258 ± 61	68	1 871 ± 84
	shade	62	93 ± 11	32	225 ± 32
**Validity period (in months)**	**Lifespan (months)**	**235**		**205**	
	[0-10[	6	1 134 ± 433	12	689 ± 99
	[10-20[	34	826 ± 121	58	967 ± 76
	[20-30[	82	493 ± 61	26	844 ± 105
	[30-40[	94	1 246 ± 87	93	1 528 ± 87
	40≤	17	1 528 ± 233	16	2 037 ± 178
	Test R		0,612		0,600
**Types of fibers**	Polyester	231	958 ± 54	191	1 264 ± 57
	Polyethylene	10	967 ± 221	15	1 468 ± 234

In rural Kribi, the use of ordinary soaps was more frequent (190/241), while in urban Kribi, the use of caustic detergents was more prevalent (124/207). Washing with ordinary soap is associated with lower pHI than washing with caustic detergents in both areas (Table V).

In urban Kribi, drying LLINs in the sun after washing was a widespread practice (68 households out of 100), while in rural areas, drying in the shade was more common (62 out of 114 households). The pHI were significantly higher for LLINs spread out in the sun than in the shade in both rural (p = 0.001) and urban (p = 0.042) Kribi. However, there is no correlation between the lifespan of LLINs (which is linked to factors such as maintenance methods, duration of use, fiber type, and manufacturer’s recommendations) and pHI at the two study sites (see Table V).

Table VI shows that mortality rates decrease with increased washing frequency of Olyset, PermaNet, and Interceptor brand LLINs in both rural and urban Kribi.

**Table VI:** Mortality rates of Anopheles gambiae (local strain) and the Kisumu strain 24 hours after exposure to 10 LLINs

	Rural Kribi					Urban Kribi	Kisumu	
Insecticides	Number of LLINs	Local strain (mortality %)	Kisumu strain (mortality %)	Brand	Number of LLINs	Local strain (mortality %)	Kisumu strain (mortality %)	Brand
**Permethrine**	**1**			**Olyset Net**	**2**			**Olyset Net**
[0-10 washes]	1	2 %	55 %		1	50 %	96 %	
[0-20 washes]	0				1	41 %	99 %	
**Deltamethrine**	**2**			**rectangular**	**1**			**rectangular**
[0-10 washes]	1	60 %	95 %		0			
[0-20 washes]	1	45 %	98 %		1	4 %	90 %	
**Alpha-cypermethrin**	**2**			**Interceptor**	**2**			**Interceptor**
[0-10 washes]	1	1 %	22 %		1	1 %	45 %	
[0-20 washes]	1	1 %	42 %		1	33 %	79 %	
**Total**	**5**				**5**		

## Discussion

LLIN technology was developed to address the low impregnation rates of conventionally treated mosquito nets [24]. The distribution and use of LLINs have become a priority for national malaria control programs [1,3]. However, promoting mosquito nets can only be effective as part of a comprehensive set of measures aimed at improving the quality of life for local populations.

In Kribi, LLIN usage rates were 73% in rural areas and 79% in urban areas. This high usage rate may be associated with the high mosquito density observed in the study area [16]. Our results are consistent with those reported in Benin and Mozambique [22,23]. According to these studies, the usage rate was 25% in Nampula and greater than 60% in Benin. Although LLIN usage is high when nets are available, actual coverage remains below the WHO-recommended threshold of ≥80%. Widespread coverage of a population (>80%) with insecticide-treated nets would provide protection even to those who do not use them [24]. It would reduce malaria transmission and incidence rates, as demonstrated by the mass introduction of lambda-cyhalothrin-treated nets in Côte d’Ivoire’s northern savanna region [15]. In order to fulfill its protective role effectively, a mosquito net must form a physical barrier that mosquitoes cannot cross. Over time, wear and tear, as well as maintenance practices such as washing frequency, type of soap used, and drying method, can impact the physical integrity of an LLI. Our study showed a positive correlation between pHI and washing frequency in rural Kribi. These results are consistent with those reported in studies conducted in central and southern Cameroon [4,7,25]. The stress placed on the fibers of the mosquito net due to frequent washing leads to the formation of Type I holes, which enlarge to Types II, III, or IV. Similar observations were made in neighborhoods in the city of Douala [19]. The type of detergent used for washing may also contribute to a mosquito net’s deterioration. In both rural and urban areas, the pHI were lower for LLINs washed with regular soap than for those washed with corrosive detergents. Dirty LLINs are soaked in detergent-containing water for several minutes or hours before washing. This washing process is thought to cause chemical interactions between the detergent molecules and the polyester or polyethylene. With bar soap, the soaking process is unnecessary, and the LLINs are washed directly. The most commonly used mosquito nets are made of polyester fiber. Polyester has many advantages over cotton, nylon, and polyethylene. It is a durable fiber that provides better ventilation and improved insecticide bioavailability. It is generally less expensive and lighter, which reduces transportation costs. Polyester also benefits from better quality control and is more popular. Community health workers should organize awareness campaigns to teach communities how to maintain LLINs.

Insecticide tests show a gradual loss of effectiveness with increased washing frequency. The Olyset Net brand is a new type of mosquito net. The insecticide permethrin (used at a concentration of 2% w/w, or approximately 900 mg/m²) is fused into a polyethylene resin fiber. These nets reportedly remain effective for three years and retain their efficacy after 30 washes [5]. However, a loss of insecticide efficacy is observed when washed with a corrosive detergent.

Permethrin and deltamethrin are unstable in basic environments. Their lifespan is significantly reduced at pH 9, but they are stable in acidic environments. Washing the mosquito net with vinegar-containing water is recommended.

For PermaNet mosquito nets, the insecticide deltamethrin (at a dose of 50 mg/m²) is mixed with a resin that coats the polyester fibers. The resin gradually releases the fixed pyrethroid into the environment. In the laboratory, after 20 washes, three minutes of contact with the treated netting still induced a 100% KD effect and 50% mortality [6]. The decline in efficacy observed after 20 washes in situ is linked to corrosive products and exposure to sunlight [24].

## Conclusion

The rural and urban populations of Kribi made extensive use of mosquito nets in 2019, at least for children. Overall, the coverage rate was high, ranging from 87% to 94%, depending on the number of sleepers per net. This exceeds the recommended rate of at least 80%. Similarly, the average number of LLINs per household was low, at 2.33 ± 0.091. Our study shows that fiber composition, washing frequency, detergents used, and drying location (shade or sun) are factors that likely affect the physical integrity and biological efficacy of a LLIN. Standardizing washing and drying procedures is difficult. The bioefficacy of LLINs against the local strain of *An. gambiae* s.l. decreased significantly, especially after repeated washings. This suggests a possible impact of pyrethroid resistance and improper maintenance practices. Raising awareness about proper maintenance and management practices may help extend the physical integrity and efficacy of LLINs.

## Informed consent and ethical considerations

Ethical approval for this study was obtained from the Faculty of Sciences at the University of Douala (UD). Administrative authorization was obtained jointly from the sub-prefects of the two districts and the prefect of the Ocean Department (Southern Cameroon) under number 497/AR/L11/A1. All study participants provided informed consent.

## Acknowledgments

We would like to thank the entire population of the Ocean Department for their cooperation.

## Funding

The authors declare that they received no funding from any organization for the completion of this work other than their own participation.

## Availability of data and materials

The datasets used and/or analyzed in this study are available from the corresponding author upon reasonable request.

## Authors’ contributions

RSM, PAN, JAMM, and PAA designed the study. RSM, MLOE, PMN, OENH, FNNT, and RN conducted the fieldwork. RSM, MLOE, PMN, MN, RN, and FNNT performed the laboratory analyses. RSM, PAA, FNNT, WDT, and MN conducted the data analysis. JAMM, PAA, and PAN supervised the study. RSM, PAN, PAA, and AN drafted the original manuscript. RSM, PNA, JAMM, AN, PAA, WDT, and EWE reviewed and edited the final version. All authors read and approved the final manuscript.

## Conflicts of interest

The authors declare no conflicts of interest.
